# Protecting Groups in Carbohydrate Chemistry: Influence on Stereoselectivity of Glycosylations

**DOI:** 10.3390/molecules15107235

**Published:** 2010-10-20

**Authors:** Jian Guo, Xin-Shan Ye

**Affiliations:** State Key Laboratory of Natural and Biomimetic Drugs, School of Pharmaceutical Sciences, Peking University, Xue Yuan Rd No.38, Beijing 100191, China; E-Mail: guojian_521@yahoo.com.cn (J.G.)

**Keywords:** protecting group, stereoselectivity, glycosylation, carbohydrate

## Abstract

Saccharides are polyhydroxy compounds, and their synthesis requires complex protecting group manipulations. Protecting groups are usually used to temporarily mask a functional group which may interfere with a certain reaction, but protecting groups in carbohydrate chemistry do more than protecting groups usually do. Particularly, protecting groups can participate in reactions directly or indirectly, thus affecting the stereochemical outcomes, which is important for synthesis of oligosaccharides. Herein we present an overview of recent advances in protecting groups influencing stereoselectivity in glycosylation reactions, including participating protecting groups, and conformation-constraining protecting groups in general.

## Abbreviations

Ac: acetyl
ADMB: 4-acetoxy-2,2-dimethylbutanoyl
Bac: bacillosamine
Bn: benzyl
BSP: 1-benzenesulfinylpiperidine
Boc: *tert*-butoxycarbonyl
Bz: benzoyl
DCM: dichloromethane
DDQ: 2,3-dichloro-5,6-dicyano-1,4-benzoquinone
DMBPP: 3-(2-hydroxyphenyl)-3,3-dimethylpropanoate
DMTM: 2,2-dimethyltrimethylene
DTBMP: 2,6-di-*tert*-butyl-4-methylpyridine
DTBS: di-*tert*-butylsilylene
FPsc: fluorous propylsulfonylethoxycarbonyl
Hex: hexyl
MP: *p*-methoxyphenyl
Msc: methylsulfonylethoxycarbonyl
NAP: 2-napthylmethyl
NIS: *N*-iodosuccinimide
Pent: *n*-pentenol
Phth: phthalimido
Pic: 2-Pyridylmethyl
PMB: *p*-methoxybenzyl
STaz: *S*-thiazolyl
TIPDS: 3,5-*O*-tetraisopropyldisiloxanylidene
TBDMS: *tert*-butyldimethylsilyl
TCP: *N*-tetrachlorophthalimido
TMBPP: 3-(2-hydroxy-4,6-dimethylphenyl)-3,3-dimethylpropanoate
Tf: trifluoromethanesulfonyl
TMS: trimethylsilyl
Tol: toluene
Troc: 2,2,2-trichloroethoxycarbonyl
TTBP: 2,4,6-tri-*tert*-butylpyrimidine

## 1. Introduction

In recent years, it has been widely recognized that oligosaccharides and glycoconjugates play important roles in diverse biological processes, including viral and bacterial infections, cell growth and proliferation, cell–cell communication, as well as immune response [1,2,3,4,5]. Studies on carbohydrates draw unprecedented attention, but still fall behind that on proteins and nucleic acids. A major obstacle is related to the fact that it is difficult to get enough and structurally well-defined carbohydrates and glycoconjugates, which often exist in nature at low-concentrations and in micro-heterogeneous forms. Chemical synthesis of oligosaccharides is one of the approaches to solve this problem.

Although great progress has been made in the construction of complex oligosaccharides [6,7,8,9,10,11,12], synthesis of oligosaccharides is still a difficult task and has not been realized in the routine automatic pattern. On the one hand, carbohydrates contain large numbers of the same functional groups, and they must be differentiated to achieve chemoselectivity and regioselectivity. On the other hand, glycosyl donors and acceptors can be connected by either α or β linkages. The anomeric mixtures cannot be directly used for biological research, and of course, this will aggravate the complications of synthesis and purification.

The stereoselective formation of glycosyl linkages is desired, but it is influenced by many factors such as leaving groups, solvents, activation systems, additives, and more importantly, protecting groups of donor and acceptor. Usually a protecting group is introduced into a molecule to temporarily mask a functional group which cannot survive the required reagents or chemical environment. Protecting groups in carbohydrates not only differentiate the same sort of functional groups to expose the one needed to be reacted, but also confer other effects to the molecules in glycosylation reactions. They can increase or decrease the reactivity and particularly, they can participate in the reactions, thus affecting the stereochemical outcomes of glycosylations.

The stereoselectivity of glycosylations is profoundly influenced by protecting groups, which has been recognized and utilized. For instance, a 2-*O*-acyl group is usually chosen for the introduction of 1,2-*trans*-glycosides whereas a 2-*O*-ether protecting group is used for the formation of 1,2-*cis*-glycosides, though often leading to glycosides with poor anomeric selectivity. Herein, we present an overview of protecting groups influencing stereoselectivity in glycosylation reactions, including participating protecting groups and conformation-constraining protecting groups in general. As there have been excellent reviews involving oligosaccharide synthesis [13,14], especially about mannosides [15], 2-amino-2-deoxysugars [16] and sialic acid-containing sugars [17,18,19,20], this review will mainly summarize the latest advances in stereoselective glycosylations from the protecting group point of view.

## 2. Participating-Type Protecting Groups

**Scheme 1:**
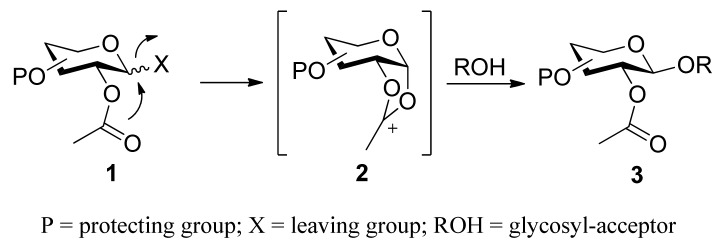
The stereoselective formation of glycosidic bond by neighboring-group participation.

Classical participating groups usually means neighboring-group participation, namely participation of acyl groups at the C2 position. During glycosylation processes, as shown in Scheme 1, the neighboring acyl group of donor **1** assists the departure of an activated leaving group, and subsequently leads to the formation of a more stable dioxolenium ion **2**. Consequently, the glycosyl acceptor can only attack from the backside to form 1,2-*trans* glycoside **3**. In this case, glucosyl-type donors give β-glycosides, while manno-type donors give α-glycosides. Many ester-type groups such as acetate, chloroacetate, benzoate, and pivaloate have been widely adopted to construct 1,2*-trans *glycosidic linkages. However, sometimes the formation of an orthoester prevents the conversion into 1,2*-trans *glycosides [21,22,23], or the removal conditions are too harsh. Moreover, classical neighboring-group participation can only be used to stereoselectively synthesize 1,2*-trans *glycosides. Recently, new participating protecting groups have been developed. The scope of participating protecting groups has gone beyond simple acyl groups. These protecting groups can be used not only to construct 1,2*-trans *glycosides, but also to construct 1,2*-cis *glycosides. Anyway, these protecting groups follow the similar mechanism--all is through “participating” with the intermediate oxocarbenium-ion, and thereby making acceptors attack only from one side.

### 2.1. Improved ester groups

**Scheme 2:**
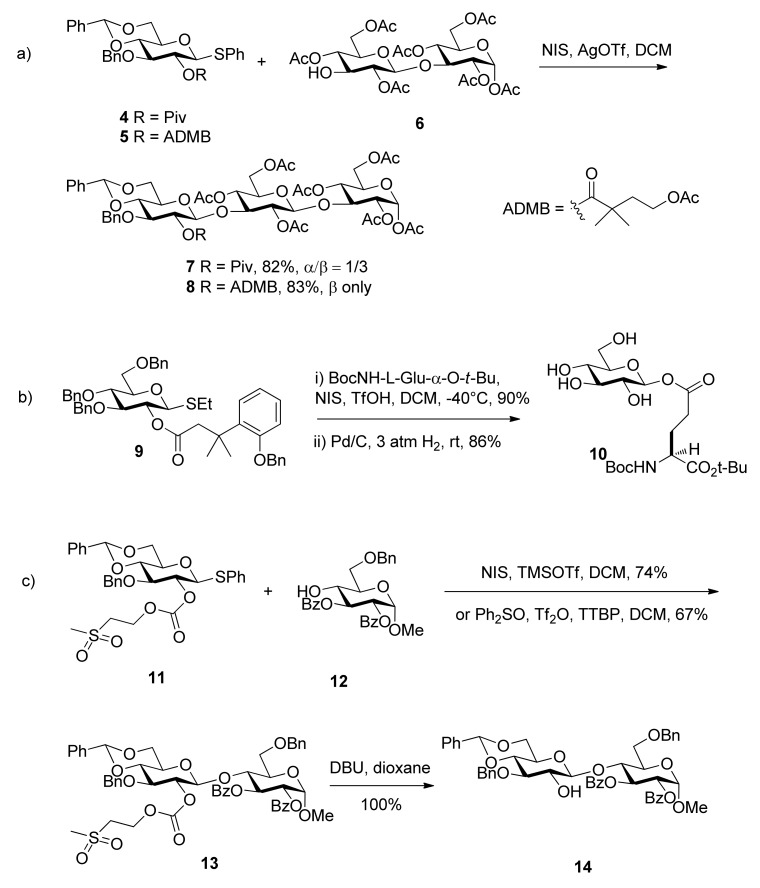
The formation of 1,2-trans glycosides directed by improved ester groups.

The 4-acetoxy-2,2-dimethylbutanoyl (ADMB) ester was reported by Ensley [24]. It showed similar selectivity to the pivaloate ester in carbohydrate acylations. When placed at the C2 position, such as in the case of compound **5**, it implemented anchimeric assistance and sterically prevented the formation of orthoesters as the pivaloate ester did. But the glycosyl donor with it showed higher β-selectivity (Scheme 2a Moreover, the removal conditions were much milder than those required for removal of the pivaloyl group. When it was cleaved, hydrolysis of the 4-acetoxy group by base initiated an intramolecular lactonization, leading to the release of stable dimethylbutyrolactone.

3-(2-Hydroxyphenyl)-3,3-dimethylpropanoate (DMBPP) and 3-(2-hydroxy-4,6-dimethylphenyl)-3,3-dimethylpropanoate groups (TMBPP) are two protecting groups that also function as described above [25]. They enable the synthesis in excellent yield of a range of β-glucosides and α-mannosides through neighboring participation. Their removal is carried out by hydrogenolysis through initiation of an intramolecular lactonization. Because these groups can be removed along with cleavage of benzyl ethers in a single reaction step and in the absence of acid or base, they are tolerant of other esters. They are particularly well suited to the stereocontrolled synthesis of glycosyl esters such as **10**, an unusual element in the fungal cell wall Pir protein from *Saccharomyces cerevisiae* (Scheme 2b).

Methylsulfonylethoxycarbonyl (Msc) was designed as a base-labile participating protecting group (Scheme 2c [26]. When situated at the O2 position of glycosyl donor **11**, the carbonate provided anchimeric assistance and was completely stable to Lewis acid-promoted glycosylations. It was cleaved by elimination using mild basic conditions under which common ester protecting groups are stable. The fluorous Msc group, fluorous propylsulfonylethoxycarbonyl (FPsc) has a similar character and can be used in a “light fluorous” trisaccharide synthesis.

All these improved ester groups mentioned above preserve the nature of esters, and lead to 1,2*-trans *glycosides when placed at a C2 position. Their improved features will make them more useful in some special cases.

### 2.2. Dialkyl phosphates

**Scheme 3:**
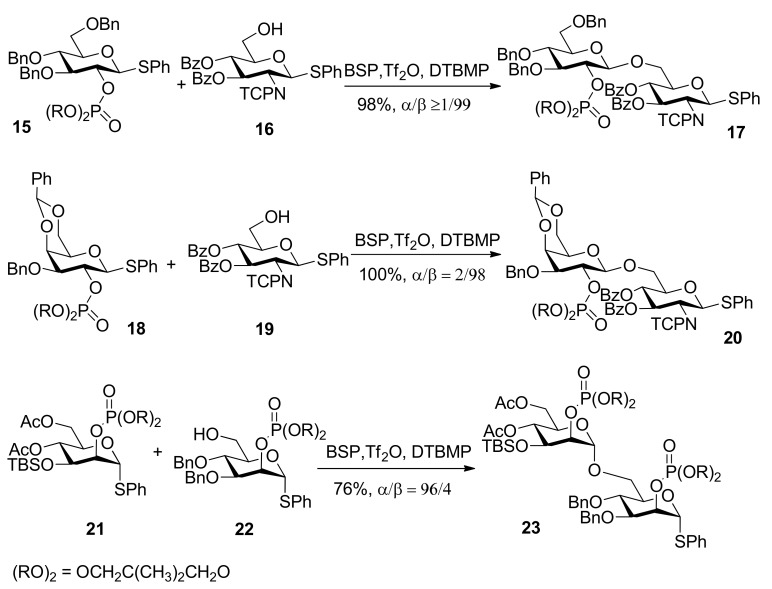
Dialkyl phosphates as stereodirecting groups.

**Scheme 4:**
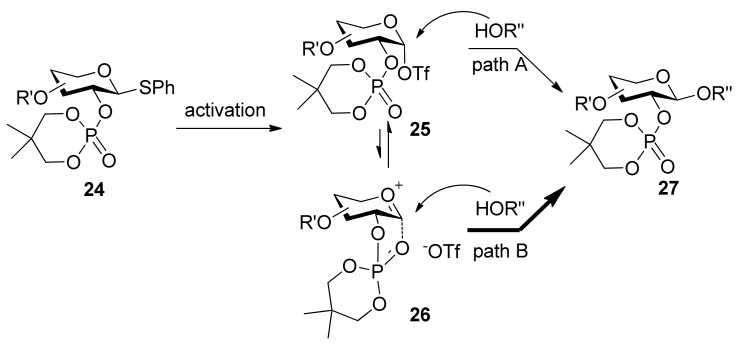
Plausible mechanism of alkyl phosphates as 1,2-*trans* stereodirecting groups.

The use of dialkyl phosphates as stereodirecting groups for the synthesis of 1,2-*trans*-glycosides was reported by Yamago *et al*. [27]. As shown in Scheme 3, the glycosylation of thioglycoside **15**, having a 2,2-dimethyltrimethylene (DMTM) phosphate group at the C2 position, provided 1,2-*trans*-glycoside **17** as the sole product under a set of glycosyl coupling conditions. Importantly, the corresponding orthophosphorate product was not detected. The DMTM phosphate group was removed in 95% yield by sodium hydroxide in a mixed solvent of ethanol/water. The coupling reactions of different glycosyl donors such as thioglycosides **18** and **21** with acceptors were examined and excellent results were obtained (Scheme 3). Although the α-glycosyl triflate **25** was detected by NMR at low temperature [28], by analysis and comparison, the authors pointed out that the neighboring participation mechanism involving intermediate **26** was more plausible and it was a good participating protecting group for the formation of 1,2-*trans* glycosides (Scheme 4).

### 2.3. 2-Pyridylmethyl group

**Scheme 5:**
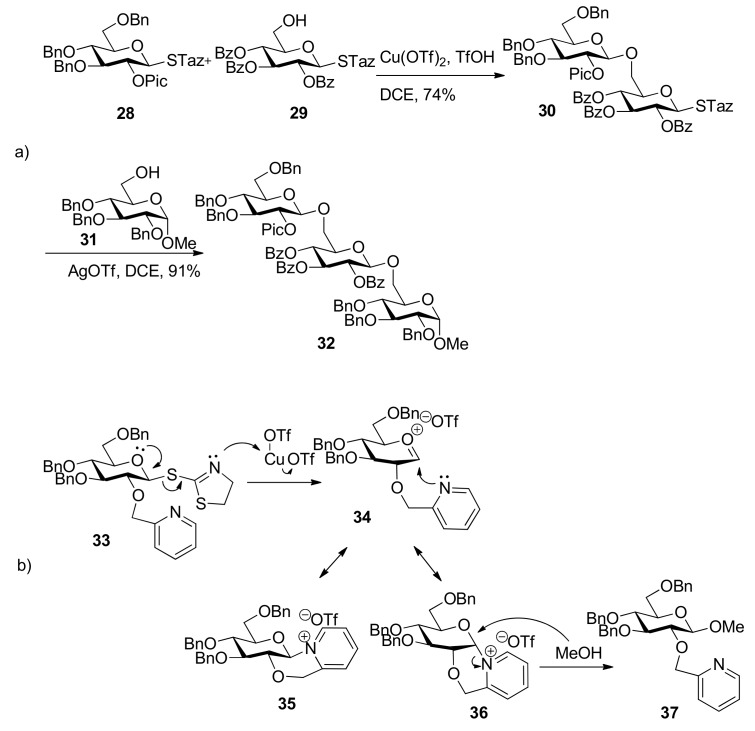
Arming participating group for stereoselective glycosylation: **a)** synthesis of a trans, trans-linked trisaccharide **32**; **b)** proposed reaction mechanism.

The Fraser–Reid armed–disarmed strategy, based on the chemoselectivity principle, offers an efficient tool for the synthesis of oligosaccharides with a *cis*, *trans*- or *cis*, *cis*-glycosylation pattern [29,30]. But generally speaking it cannot be applied to the synthesis of *trans*, *cis*- or *trans, trans*-linked oligosaccharide fragments. The 2-pyridylmethyl group was designed as “arming participating group” to extend the scope of armed–disarmed strategy and allowed stereoselective introduction of a 1,2-*trans* linkage prior to another 1,2-*trans* or 1,2-*cis* linkage (Scheme 5a [31]. This protecting group is capable of efficient participation through a six-membered intermediate **36 **(Scheme 5b) as acyl participating group to show complete 1,2-*trans* selectivity, but it belongs to the ether protecting groups that are capable of activation to retain the glycosyl donor in the armed state as opposed to conventional acyl participating moieties. The β intermediate **35** remained completely inert and could be isolated from the reaction mixture, so the corresponding 1,2-*cis* glycosides were not produced. With this expansion, the armed–disarmed approach would theoretically allow convergent chemoselective synthesis of any oligosaccharide sequence.

### 2.4. Chiral auxiliary groups

**Scheme 6:**
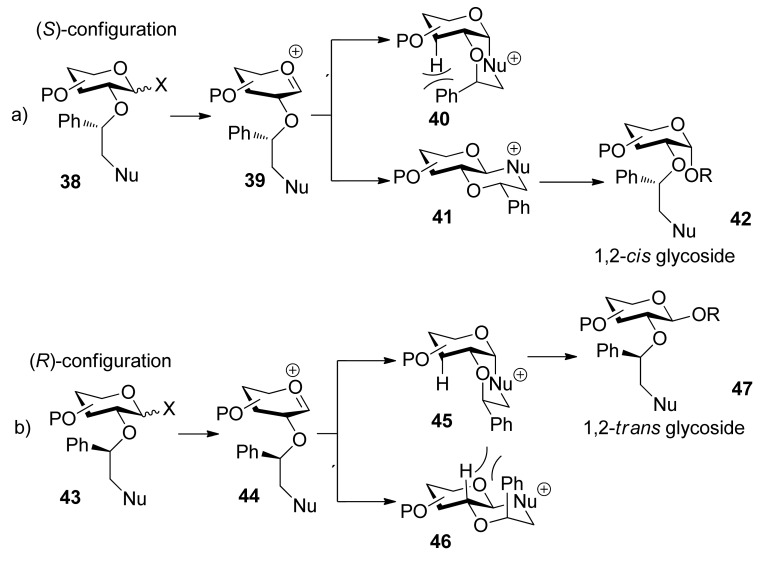
The mechanism of chiral auxiliary group participating.

In 2005, Boons and coworkers reported a novel strategy for stereoselective glycosylations in which a chiral auxiliary at the C2 position of a glycosyl donor was used (Scheme 6) [32]. The auxiliary is a substituted ethyl moiety that contains a nucleophilic group. Upon formation of an oxocarbenium ion, participation of the nucleophilic moiety of the auxiliary should lead to the formation of either a *cis*- or a *trans*-decalin system (**40, 45** or **41, 46**), determined by the configuration of chiral auxiliary at the C2 position. Because of steric repulsion between the phenyl group and the proton, the auxiliary with *S* stereochemistry would lead to the formation of 1,2-*cis* glycosides via the *trans*-decalin intermediate **41**, whereas the auxiliary with *R* stereochemisty would lead to the formation of 1,2-*trans* glycosides via the *cis*-decalin intermediate **45**.

**Scheme 7:**
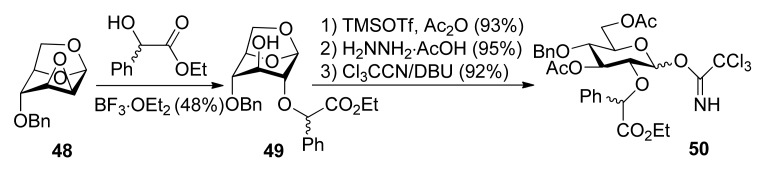
Preparation of donors having auxiliary ethyl mandelate.

**Scheme 8:**
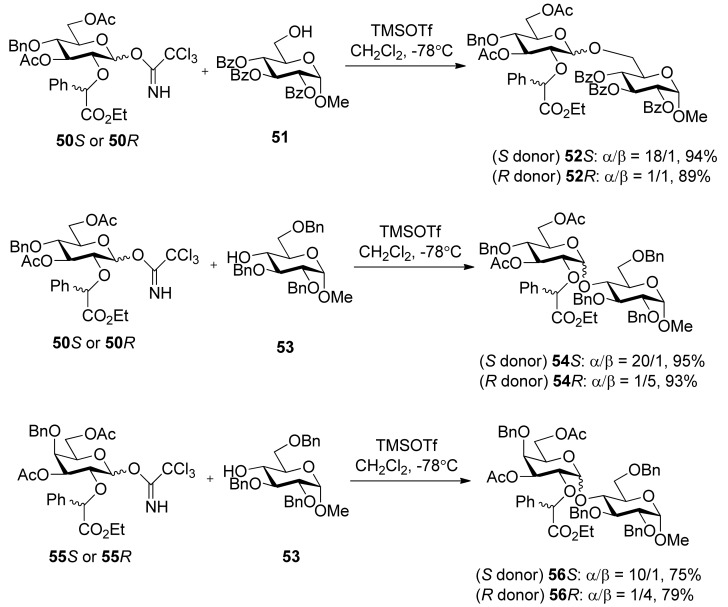
Stereoselective glycosylations with glycosyl donors **50** and **55**.

Ethyl mandelate was explored as the first-generation chiral auxiliary, because of its ready availability, appropriate participating ester functionality, and ready removal under mild reductive conditions like the benzyl group. Glucosyl donors **50***R* and **50***S*, containing a (*R*) or (*S*)-ethoxy-carbonylbenzyl moiety, were prepared from epoxide **48** and ethyl (*R*) or (S)-mandelate (Scheme 7). Glycosylations of **50***S* with glycosyl acceptors that have either a primary hydroxyl group or a secondary hydroxyl group exposed yielded disaccharides with high α-anomeric selectivity (Scheme 8). Correspondingly, the use of glycosyl donor **50***R* led to a reversal of anomeric selectivity and mainly afforded β-linked disaccharides. This methodology was also applied to galactosyl donors **55***S* and **55***R* [33]. It was observed that the α-anomeric selectivity was high for the donor with *S* chiral auxiliary, however, the β-anomeric selectivity was low for the donor with *R* chiral auxiliary.

**Scheme 9:**
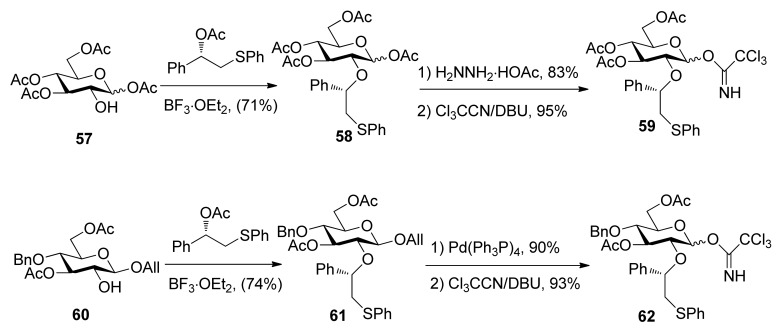
Preparation of donors having auxiliary (*S*)-(phenylthiomethyl)benzyl group.

**Scheme 10:**
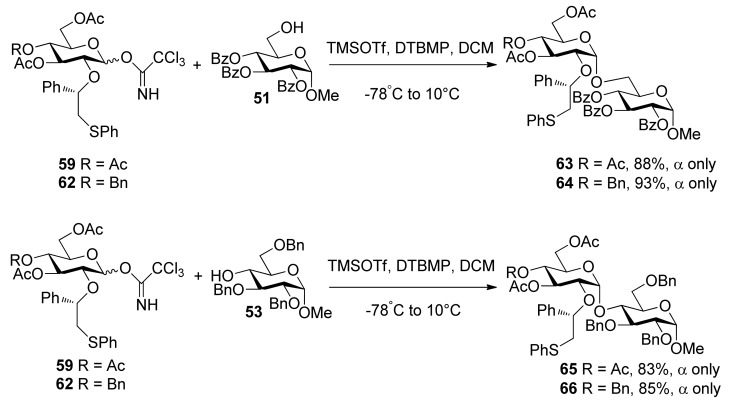
Stereoselective glycosylations with glycosyl donors **59** and **62**.

**Scheme 11:**
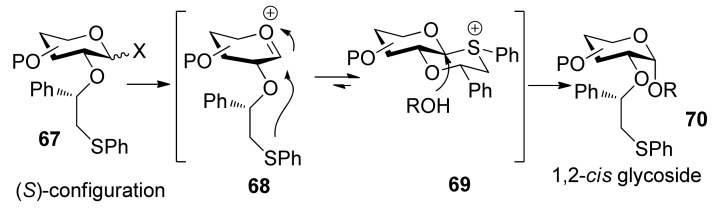
The mechanism of (*S*)-(phenylthiomethyl)benzyl moiety participation.

To consummate the methodology, the second-generation chiral auxiliary with a (*S*)-(phenylthio-methyl)benzyl moiety was developed [34]. Donors **59** and **62** were easily prepared following the synthetic route shown in Scheme 9. The coupling of donors **59** and **62 **with a range of glycosyl acceptors all gave only α-anomers with good yields (Scheme 10), which showed the validity and generality of the approach. The fact that the glycosylations lead to the formation of exclusively α-anomers provides support that the reactions proceed through an equatorially substituted anomeric sulfonium ion **69**, the presence of which was confirmed by low-temperature NMR spectra. It is believed that a combination of an equatorially oriented (1*S*)-phenyl substituent and *trans*-decalin formation is important feature for controlling the α-anomeric selectivity (Scheme 11). On the other hand, worse than the donor with ethyl (*R*)-mandelate (**50***R*), glycosylations with the donor having (*R*)*-*(phenylthiomethyl)benzyl at O-2 position resulted in anomeric mixtures with no selectivity.

**Scheme 12:**
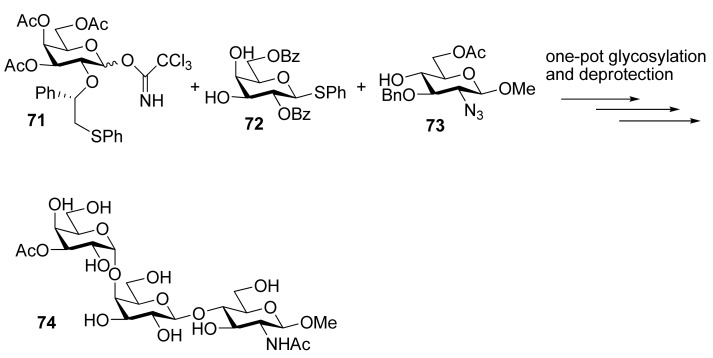
One-pot two-step synthesis of trisaccharide **74**.

**Figure 1:**
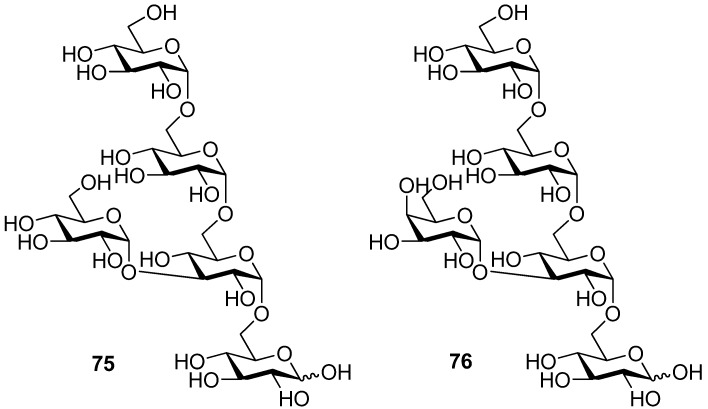
Branched oligosaccharides **75** and **76**.

To demonstrate the combined use of this method and the classic neighboring-group participation, trisaccharide **74**, an epitope that can trigger acute rejections in xenotransplantations [35], was synthesized from galactosyl donor **71 **using a one-pot two-step glycosylation procedure (Scheme 12). Recently, this methodology was also successfully used for the solid-supported synthesis of complex branched oligosaccharides **75** and **76** [36], a repeating unit of α-glucan pentasaccharide found in *A. carmichaeli* [37] (Figure 1). Multiple 1,2-*cis*-glycosidic linkages were introduced by using glucose and galactose donors having a participating (*S*)-(phenylthiomethyl)benzyl chiral auxiliary at O2 position, with complete anomeric control. These successful examples demonstrate that the methodology is compatible well with the solid-phase synthesis.

As novel stereodirecting groups, chiral auxiliary groups could be successfully used to construct 1,2-*cis*-glycosides by “participating” with the intermediate oxocarbenium-ion. Inefficiency for introduction of 1,2-*trans*-glycosides is their drawback, however, classical neighboring group participation using ester protecting groups can deal with that easily. The combination of them will make stereoselective oligosaccharide synthesis more efficient. 

### 2.5. Remote participation groups

Some protecting groups can use remote participation to control the configuration of the anomeric center in glycosylation reactions [38,39,40,41,42]. Because their roles are sometimes ambiguous and controversial, it is hard to say what these groups are, or when they function. Here are some recent examples, some of which are specifically designed to check whether or not remote participation plays a role in glycosylation reactions. The resolution of these issues is helpful in the development of stereocontrolled oligosaccharide synthesis.

**Scheme 13:**
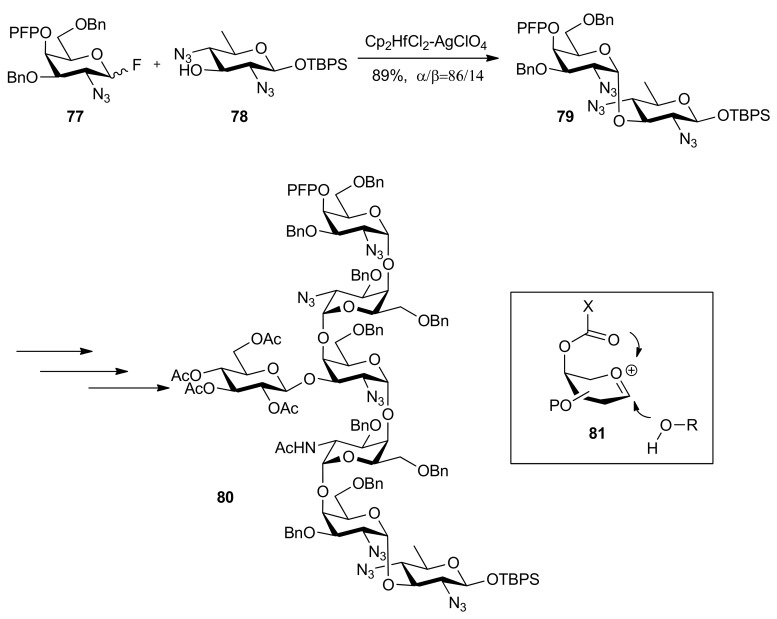
Synthesis of a heptasaccharide from Gram-negative bacterium.

High α-anomeric selectivity could be gained by remote participation in 1,2-*cis*-galactosylation [43]. Ito and co-workers reported the synthesis of *N*-linked glycan derived from Gram-negative bacterium, *Campylobacter jejuni,* a heptasaccharide composed of Asn-linked bacillosamine (Bac), repeating *N*-acetylgalactosamine, and branching glucose [38]. This synthesis started from the Bac-acceptor **78**, which was consecutively glycosylated with 4-*O*-pentafluoropropionyl (PFP) protected galactose donors such as **77**, finally affording the heptasaccharide **80 **(Scheme 13). The function of 4-*O*-pentafluoropropionyl group was considered as not only temporary protection but also remote participation from β face, which made the galactosylation proceed in a highly α-selective manner. In addition, this protecting group could be removed under extremely mild conditions, even with *O*-Ac groups completely intact [44].

**Scheme 14:**
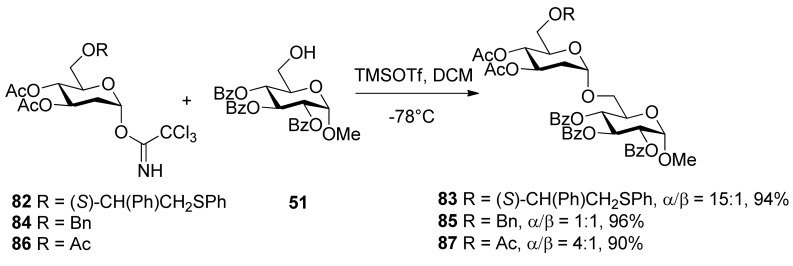
Synthesis of 2-deoxyglycosides using donors with different protecting groups at O6 position.

α-Linked 2-deoxyglycosides were exclusively prepared by employing glycosyl donor 82 having a participating (*S*)-(phenylthiomethyl)benzyl moiety at O6 position (Scheme 14) [45]. Similar glycosylations employing glycosyl donors 84 and 86, having a benzyl ether or acetyl ester at the C6 position, provided the disaccharides 85 and 87, respectively, as mixtures of anomers. The use of (*R*)-(phenylthiomethyl)benzyl ether at C6 position of the glycosyl donor also led to excellent anomeric selectivity, indicating that the chirality of the auxiliary does not influence the anomeric outcome of glycosylations. Although the intermediate sulfonium ion was not identified by NMR, it was thought that the remote participation of (phenylthiomethyl)benzyl moiety is a key factor.

**Scheme 15:**
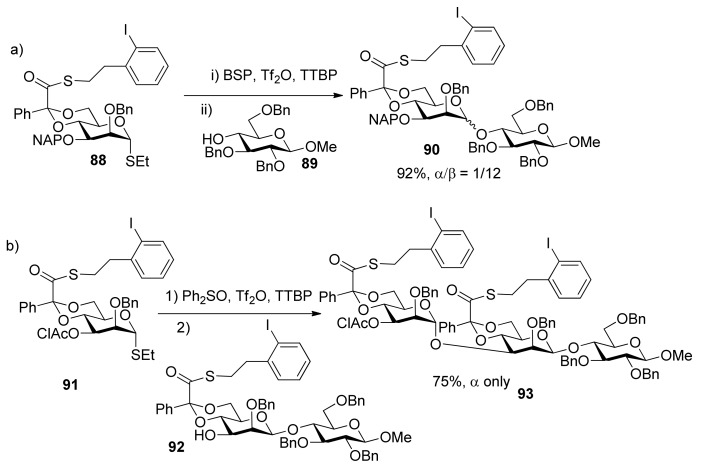
Comparison of influence of different groups at C3 on stereoselectivity.

**Scheme 16:**
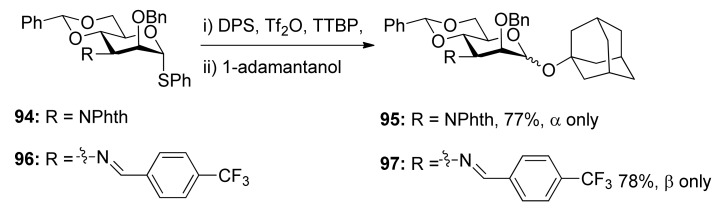
Comparison of influence of different groups at C3 on stereoselectivityin the synthesis of 3-amino-3-deoxy-mannopyranosides.

In stereocontrolled synthesis of β-rhamnosides from mannosyl glycosyl donors [46], although the 4,6-*O*-benzylidene acetal and 3-*O*-ether group are strong control elements permitting introduction of the β-mannopyranosides [47,48,49] such as in the synthesis of **90**, the 3-*O*-chloroacetate of donor **91** was found to be strongly α-directing and to overcome the β-directing influence of the 4,6-*O*-benzylidene acetal group (Scheme 15). 3-*O*-Benzoyl group had the similar function [50]. A similar situation happened in the synthesis of 3-amino-3-deoxy-β-mannopyranosides (Scheme 16) [51]. Obviously, 3-*O*-acyl groups in 4,6-*O*-benzylidene-protected manno-type donors resulted in α-selectivity. It is likely that remote participation of acyl groups could account for the reversion of stereochemical outcomes.

**Scheme 17:**
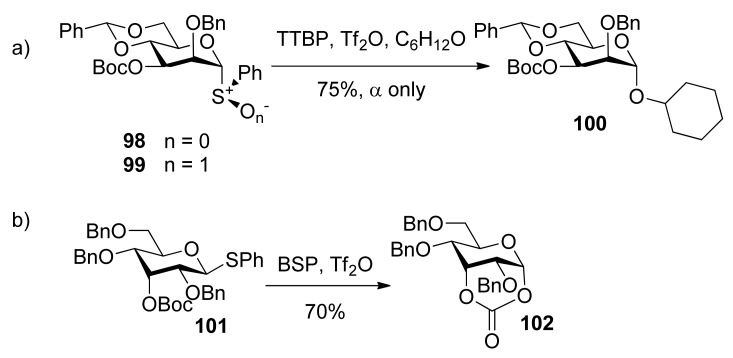
Remote participation by non-vicinal esters.

To check whether the remote participation by non-vicinal esters plays a role in glycosylation reactions, donors **98** and **99** with 3-*O*-Boc esters were designed to trap anomeric oxocarbenium ion intermediates (Scheme 17) [52]. Although only α product was obtained, formation of a cyclic carbonate spanning positions 1 and 3 of the pyranose ring was not detected. However, in another case, the axial 3-*O*-ester in allose donor **101** was able to trap anomeric oxocarbenium ion intermediate to form cyclic carbonate **102**, showing that the remote participation was unambiguous in the course of glycosylation reactions.

**Scheme 18:**
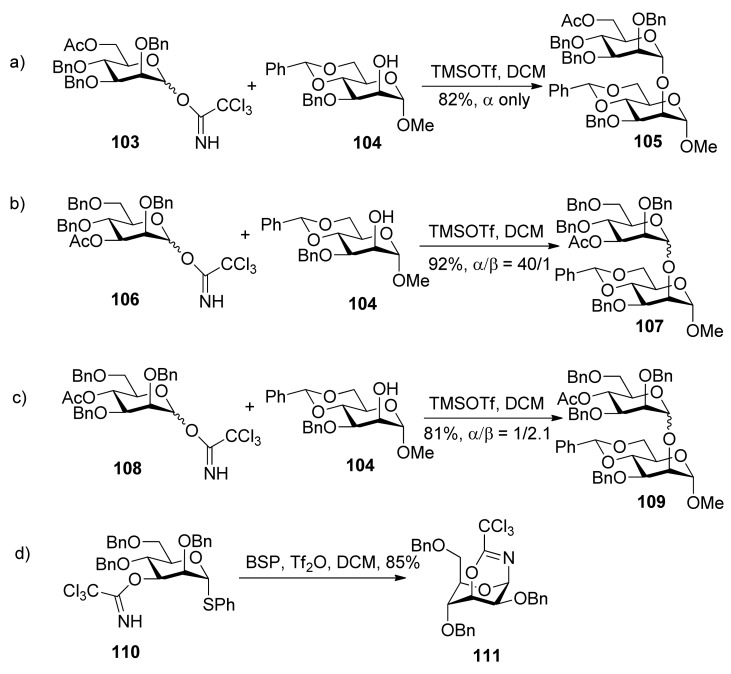
The comparison of α-directing effect with *O*-acyl group at different positions in mannopyranosylations and trapping of the intermediate.

Kim reported α-directing effect by remote participation of 3-*O*-acyl and 6-*O*-acetyl groups of donors in mannopyranosylations [53]. As shown in Scheme 18, donors **103** and **106 **showed α-selectivity, but donor **108** did not. To confirm whether or not remote participation functions, they also designed experiments to trap anomeric oxocarbenium ion intermediates by the intramolecular nucleophilic attack of the *tert*-butoxycarbonyl or the trichloroacetimidoyl group at the O3, O4, or O6 positions of mannosyl donors. They obtained stable bicyclic product **111**, having a six-membered trichlorooxazine ring, in 85% yield. 

**Figure 2:**
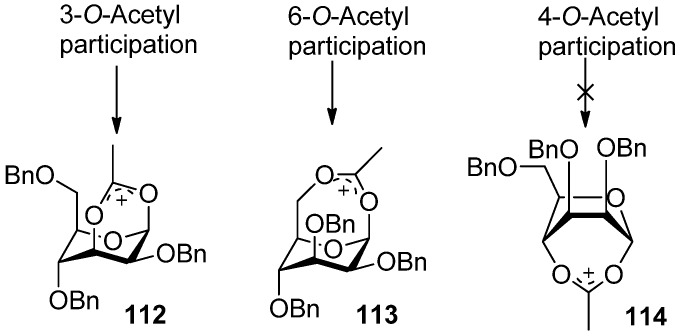
Dioxocarbenium ion intermediates by remote participation.

^1^H-NMR spectral data clearly indicated that the 3-trichloroacetimidate **110 **was in the ^4^*C*_1_ conformation, while the sugar ring of the bicyclic product **111 **was in the ^1^*C*_4_ conformation. Bicyclic products with a seven-membered trichlorooxazepine ring corresponding to the trichloroacetimidoyl group at the O4 or O6 positions of mannosyl donors were not isolated. Nevertheless, a small amount of a bicyclic product corresponding to the trichloroacetimidoyl group at the O6 position was detected in the product mixture by mass spectrometry. This demonstrated the remote participation by 3-*O*-acyl and 6-*O*-acetyl groups may function, but no participation by 4-*O*-acyl group worked. This could be explained by comparing the relative stabilities of dioxocarbenium ion intermediates (**112**>**113**>>**114**) (Figure 2). The more stable the bicyclic dioxocarbenium was, the stronger the participation of acyl groups was, which led to higher α-directing effect.

## 3. Conformation-Constraining Protecting Groups

Conformation-constraining protecting groups here mainly refer to cyclic bifunctional protecting groups [54], including benzylidene, carbonate, oxazolidinone, cyclic silyl groups, and others. It was thought that they can restrict the flexibility of sugar rings, favor a certain conformation of the intermediate, and thus making the glycosyl intermediate to be accessed more easily from one side. The adoption of these groups has gained great achievements in stereoselective glycosylation reactions.

### 3.1. Benzylidene group

**Figure 3:**
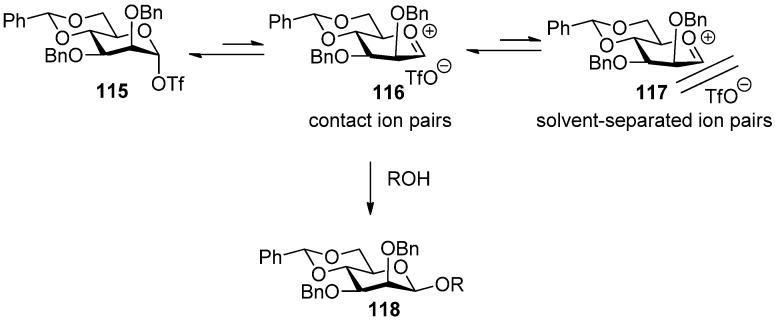
Proposed mechanism for 4,6-*O*-benzylidene-directed β-mannosylation.

Crich *et al.* developed an attractive approach to construct the β-mannosidic linkage by the *in situ* formation of α-triflate or the transient contact ion pair derived from it [28,48,49,55,71]. In this methodology, a benzylidene group was proven to be crucial for the high β selectivity. The proposed mechanism was shown in Figure 3. 

It was proposed that the benzylidene protecting group opposes the oxacarbenium formation, which leads to *S*_N _1 replacement and anomeric mixtures, due to the torsional strain engendered by the half chair or boat conformation of the intermediate, but favors the formation of an α-triflate intermediate. Speaking of electronic effects, the benzylidene group locks the C5-C6 bond in the *trans*-gauche (tg) conformation in which the C6-O6 bond is held antiperiplanar to the C5-O5 bond, thereby maximizing its electron-withdrawing effect of oxacarbenium ion and favoring its transformation to the α-triflate intermediate [56]. The α-triflate intermediate **115** was quite stable at low temperature and acted as a reservoir for a transient contact ion pair **116**. The transient contact ion pair **116** was the actual glycosylating species in the β-mannosylation reaction and was determined by α-deuterium kinetic isotope effects [55]. Because of the dynamic equilibrium between the α-covalent triflate and the contact ion pair, the closely associated triflate counterion shielded the α-face and the *S*_N_2-like substitution occurred, leading to the formation of β-mannosides. In order to form the intermediate α*-*triflate to control stereoselectivity, donors usually should be preactivated [57] by activator before acceptors were added. If donors and acceptors were added together at the same time, β-selectivity would not be observed. A more detailed mechanism was also reviewed recently by Crich [58].

**Scheme 19:**
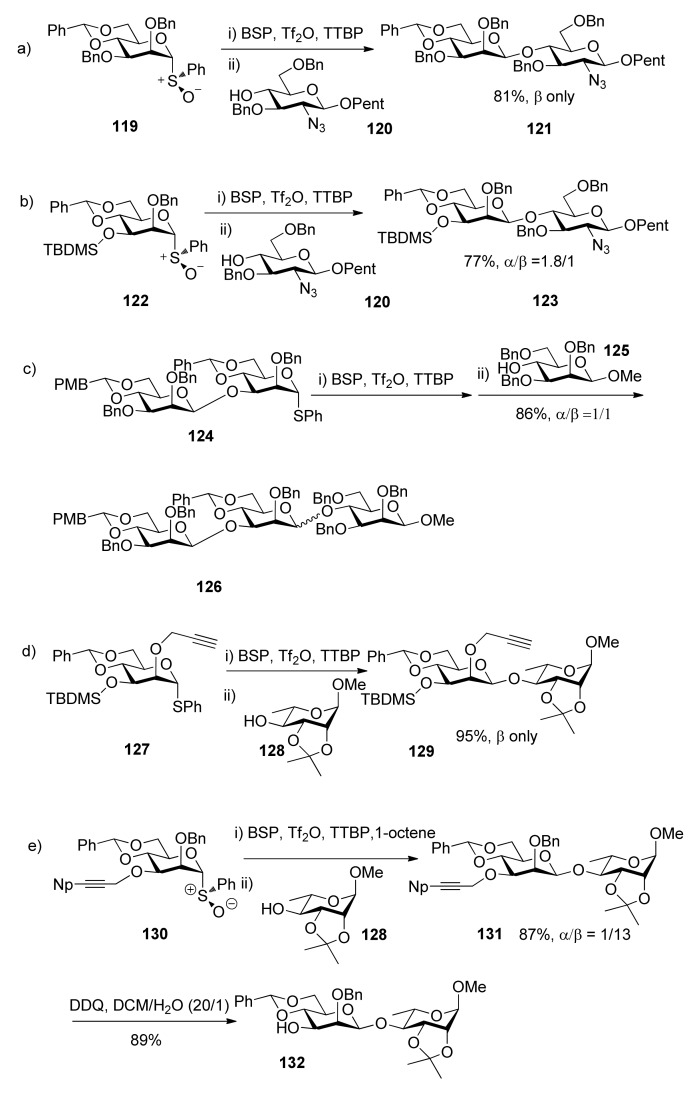
β-Mannosylation reactions with 4,6-*O*-benzylidenated mannosyl donors.

Recently, 4,6-O-benzylidenated mannosyl donors with different leaving groups and protecting groups were checked in β-mannosylation reactions [51,59,60,61,62,63,64]. It was suggested that protecting groups at the C2 and C3 positions are also be of critical importance. Usually O-benzyl ethers at C2 and C3 such as donor **119 **can assure good β-selectivity (Scheme 19a-orientated convergent oligosaccharide synthesis orthogonal protection of the two hydroxyl groups is needed. In this situation, non-participating protecting groups that have the approximate steric bulk of benzyl ethers on both at C2 and C3 are required [60,65,67]. Presumably the O2-C2-C3-O3 torsional interaction is the key factor for β-selectivity in this system. Only a proper O2-C2-C3-O3 torsion angle leads to good β-selectivity. Larger or smaller steric bulk than benzyl ethers on both at C2 and C3 would make torsional interaction deviate from the optimal value and have an influence on the equilibrium in Figure 3, thus reducing selectivity. As a result, protecting groups here must be compromised as viewed from steric bulk for good β-selectivity. When bulky silyl groups or glycosidic linkages were located at O3 position, such as in the case of donors **122** and **124**, poor selectivity was observed, even with 2-*O*-benzyl ether (Scheme 19b and 19c), but sterically minimal propargyl ether or 4-trifluoromethylbenzenepropargyl ether are good choice (Scheme 19d) [60,66,67]. When benzyl ether was placed at C2, the corresponding 3-O-propargyl donor showed poor β-selectivity but the corresponding 3-O-1-naphthylpropargyl donor **130** showed good β-selectivity (Scheme 19e) [59]. In addition, it was confirmed that ester groups at O3 could overcome the β-directing influence of the 4,6-*O*-benzylidene acetal and were strongly α-directing, as mentioned above.

**Scheme 20:**
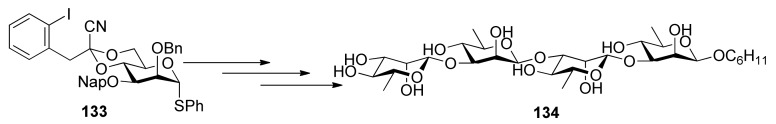
β-Rhamnosides prepared from 4,6-*O*-benzylidenated mannosyl donors.

As stereochemical analogs of β-mannopyranosides, β-rhamnosides can be synthesized from mannosyl glycosyl donors such as **133** (Scheme 20). By using functionalized benzylidene group, β-mannopyranosides can be constructed stereoselectively, which can be converted to β-rhamnosides by deoxygenation at C6 position after glycosylation [68,69].

Except for 4,6-*O*-benzylidenated mannosyl donors, 4,6-*O*-benzylidenated glucopyranosyl donors [70,71] and galactosyl donors [72] were also checked, but α*- *rather than β*-*anomeric selectivity was achieved.

### 3.2. Carbonate and oxazolidinone groups

Inspired by 4,6-*O*-benzylidene-directed mannosylation, Crich reported the synthesis of β-glucosides using carbonates as conformation-constraining protecting groups [73]. Its mechanism of the influence on stereoselectivity can be explained as the same as that of benzylidene. 

**Scheme 21:**
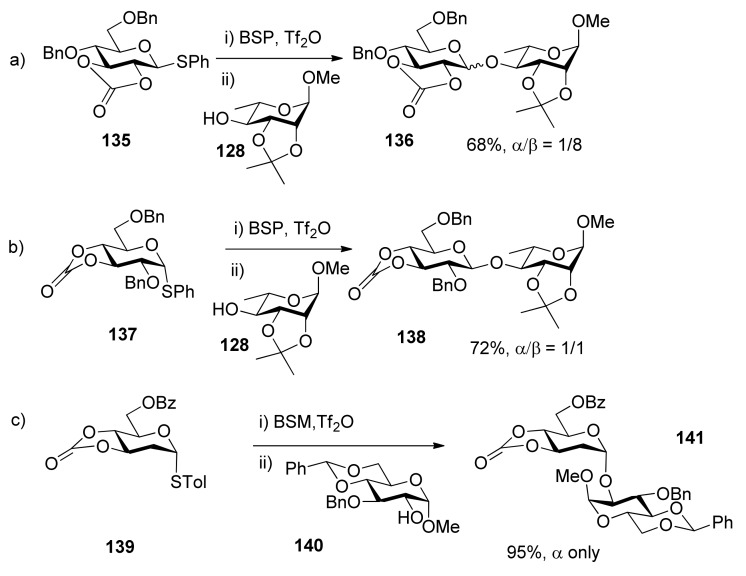
Glycosylation reactionsof carbonate-protected donors.

In donor **135**, the 2,3-*O*-carbonate group is *trans*-fused, and it opposes the formation of oxocarbenium ion, but favors the formation of an α*-*triflate intermediate, no matter whether we consider conformation or electronic factors. So donors with 2,3-*O*-carbonate groups provide a means for the formation of 1,2-*trans*-equatorial glycosidic bonds without recourse to neighboring group participation and the associated orthoester formation. In contrast, the 3,4-*O*-carbonated donor **137** shows week β*-*selectivity because it is more far away and has less influence on the anomeric center. Recently, our group reported an efficient method for the highly α-stereoselective glycosylation of 2-deoxysugars and 2,6-dideoxysugars using 3,4-*O*-carbonated donors (Scheme 21c) [74]. Compared with the corresponding peracetyl-protected donors [75], 3,4-*O*-carbonated donors showed better α*-*directing effects. Aside from the electronic effect, the conformation-constrained by carbonate may be another key factor, which could lead to *S*_N_2-like displacement, possibly through the more active β*-*triflate intermediates. Although carbonate-directing selectivity as described above may result from α*- * or β*-*triflate intermediates, it has not yet been proven by experiments.

**Scheme 22:**
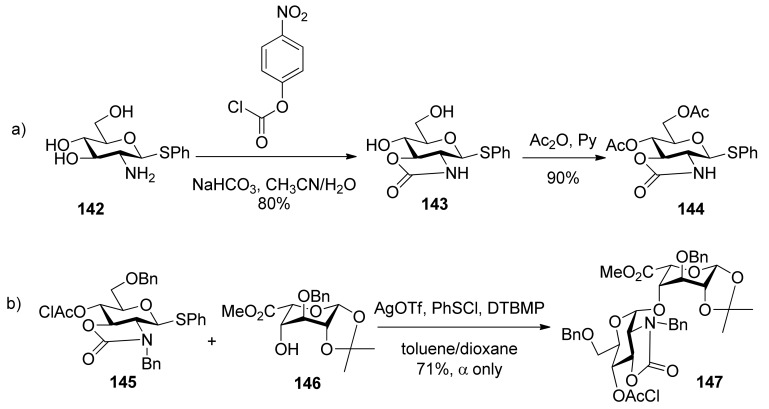
The preparation and glycosylation reactions of oxazolidinone-protecting donors.

**Figure 4:**
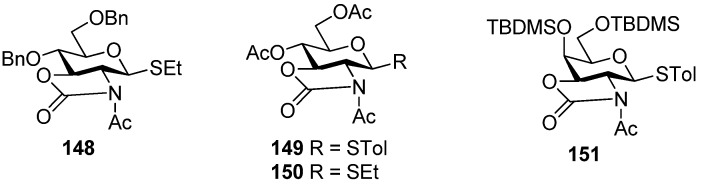
*N*-Acetyl 2,3-*trans*-oxazolidinone-protected donors **148-151**.

Much attention has been paid to the oxazolidinone group because it is very useful in the synthesis of α-2-amino-2-deoxyglucopyranosides and α-sialosides. The standard conditions employed for the introduction of the oxazolidinone group are the use of 4-nitrophenyl chloroformate (Scheme 22a [76], or the improved protocol with triphosgene [77]. Usually,an oxazolidinone group as non-participating group at C2 can facilitate the simultaneous differentiation of the 2-amino and 3-hydroxyl from other hydroxyl groups, and plays a role via a conformation-constraining mechanism. Initially, 2,3-oxazolidinone-protected thioglycoside donors, such as **144**, were explored [76,78]. Although excellent α-selectivity was achieved, the obvious drawbacks included difficult activation and *N*-glycosylation—the nitro atom reacted as a nucleophile. To overcome these shortcomings, *N*-protected donors were developed. *N*-Acetyl protected glucosamine donors **148**, **149**, and **150** (Figure 4) showed convertible selectivity by tuning the reaction conditions [77,79,80,81,82]. *N*-Acetyl protected galactosamine donor **151** was used to synthesize repeating α-(1→4)-linked *N*-acetyl-galactosamine units and showed good α*-*selectivity [83].*N*-Benzyl 2,3-*trans*-oxazolidinone-protected donor **145 **showed high α*-*selectivity for secondary alcohol, while for primary alcohol, high α*-*selectivity was also obtained but by virtue of solvent effects [84]. Concerning the mechanism involved, Oscarson made an investigation on *N*-acetyl protected glucosamine donor **150** using NMR-monitored glycosylation and anomerization experiments. It was suggested that a β-linkage was exclusive formed initially, but in the presence of the oxazolidinone group, β-glycosides anomerized to the α-glycosides through an intramolecular mechanism involving an endocyclic C-O bond cleavage [82]. Other oxazolidinone-protected donors maybe follow the same mechanism.

**Scheme 23:**
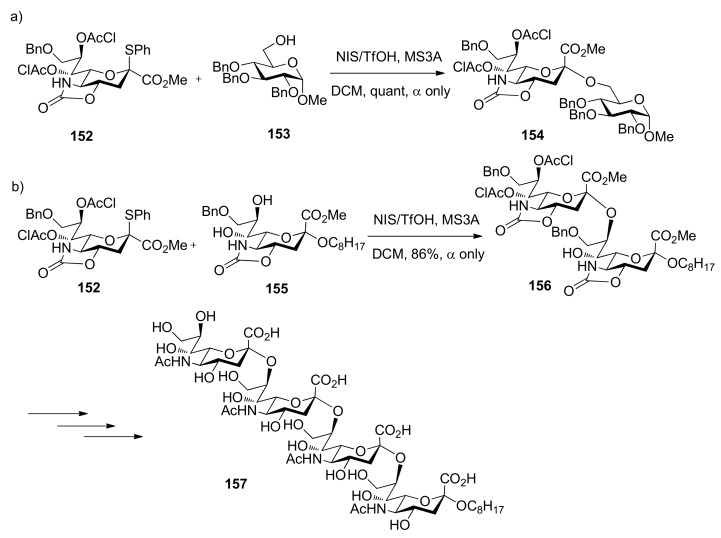
α-Sialylation using the oxazolidinone-protected donor.

The oxazolidinone group was also successfully used in α-sialylation reactions. Takahashi and co-workers reported sialylation reactions using 5-*N*,*4*-*O*-carbonyl-protected sialyl donor **152 ** [85]. This donor with 4,5-oxazolidinone and 7,8-*O*-dichloroacetyl groups showed not only high reactivity but also high α-selectivity. The α-sialylation product could be obtained quantitatively using donor **152** and acceptor **153** without assistance of nitrile solvent. Biologically important α(2,8) and α(2,9) polysialic acids such as **157** could also be prepared using this methodology (Scheme 23) [85,86]. However, the mechanism for this unique α-sialylation was not examined in their work. 

**Scheme 24:**
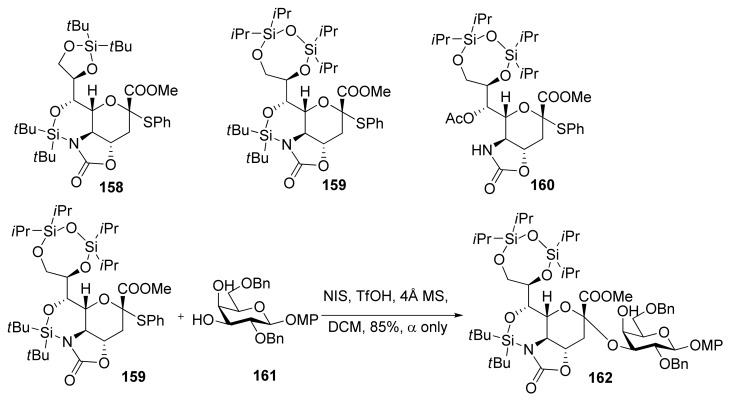
The silylene and oxazolidinone double-locked donors and α-sialylation with them.

**Figure 5:**
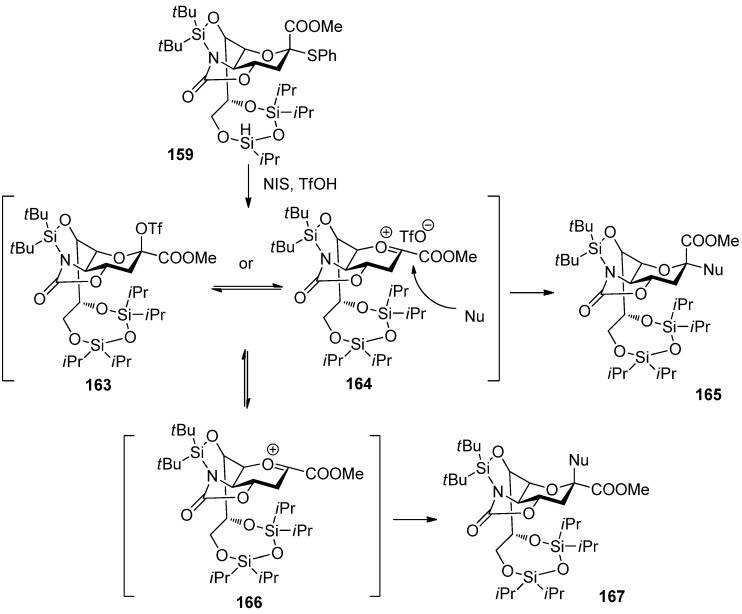
Plausible reaction mechanism of the donor **159**.

Crich made a comparison study between *N*-acetyl-5-*N*,4-*O*-carbonyl-protected sialyl donor and *N,N*-diacetyl sialyl donor, and showed the former exhibited superior yields and α-selectivity, which proved oxazolidinone group had positive influence on α-sialylation in this system [87]. Recently, Hanashima reported a comparison study of silylene and oxazolidinone double-locked sialyl donors [88]. Donors **158 **and **159**, which had an additional C5,7-DTBS lock, showed comparable α-selectivity with donor **160** when coupled with the C6-OH group of galactose acceptors. When the double-locked donors, especially **159**, were subjected to sialylation with the C3-OH group of galactosides such as **161**, they showed much better α-selectivity than **160 **(Scheme 24). It was proposed that the silylene and oxazolidinone double-lock in **158 **and **159 **would stabilize the conformation of the pyranose ring more than the single oxazolidinone-type donor **160 **(Figure 5). Such conformational constraints would stabilize plausible intermediates **163**/**164**. For acceptors with high reactivity, such as primary alcohols and **161**, the hydroxyl group would attack from the α-face of intermediates **163**/**164 **through an S_N_2-like pathway to afford the α-anomers. 

In contrast, acceptors with low reactivity would find it difficult to react with relatively stable intermediates **163**/**164**. Thus, **163**/**164** would decompose to give highly reactive oxonium ion **166**, which could react with acceptors from the β-face to afford the β-anomers. Except these examples mentioned above, protecting groups at C5 of sialyl donors usually show a profound influence on the selectivity and reactivity of α-sialylation [89,90], which has been well summarized in other reviews [17,19].

### 3.3. Cyclic silyl groups

**Scheme 25:**
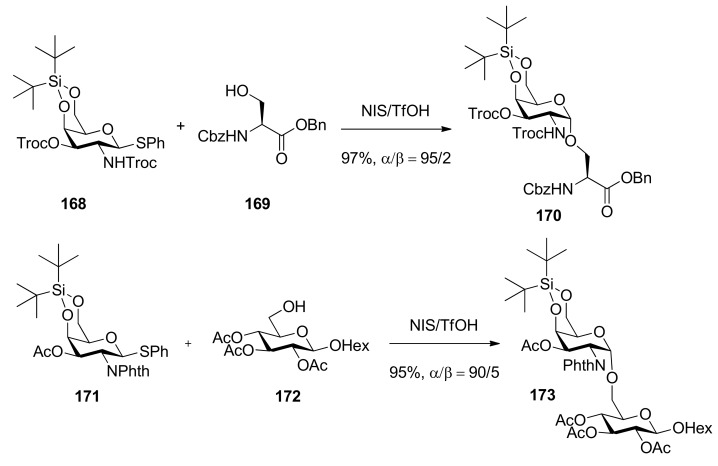
The glycosylation reaction with the donor having di-*tert-*butylsilylene.

Di-*tert*-butylsilylene (DTBS)-directed α-galactosylation was reported by Kiso [91,92]. In these works, both galactosaminyl and galactosyl donors were examined extensively. It was suggested that this method was compatible with a wide variety of leaving groups and common ether and ester protecting groups, even participating groups on C2 oxygen or nitrogen such as benzoyl, Troc, and Phth groups, and all gave α-predominant products (Scheme 25), except that it showed reversal selectivity when insoluble silver silicate was selected as a promoter for the corresponding glycosyl bromide donor. 

**Figure 6:**
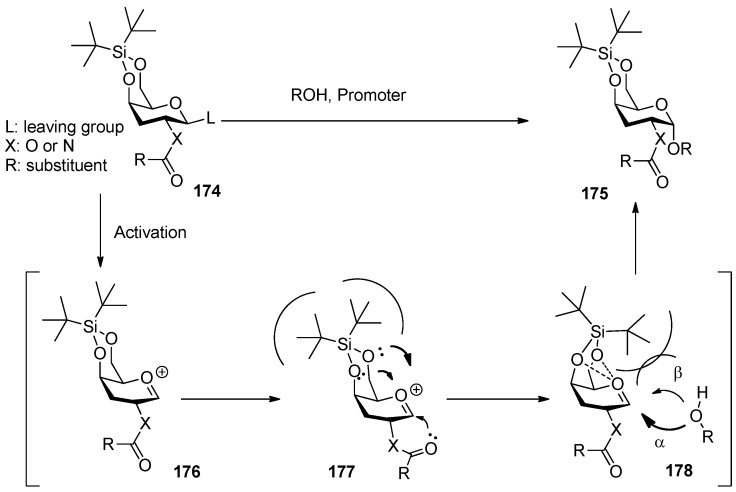
Proposed reaction mechanism for the DTBS-directed α-galactosylation.

The X-ray crystallographic analysis of the donor **171** showed a six-membered ring comprised of a 4,6-*O*-DTBS acetal moiety and a C4-C5-C6 bond adopted near half-chair conformation, which resulted in the *tert*-butyl group being positioned closer to the anomeric carbon. This implies that maybe the steric effect of the DTBS group leads to the α-selectivity. Further explanation is as follows: “through-space electron donation” may account for the enormous α-selectivity resulting from the DTBS effect [93,94] (Figure 6). Because of the strong stabilization of the oxocarbenium ion by axially oriented C4 electronegative substituent (through-space electron donation), participating groups on C2 oxygen or nitrogen could not participate with the intermediate oxocarbenium-ion effectively, together with steric effect of the DTBS group, resulting in the high α-selectivity. Recently, the synthesis of novel GM2 analogues based on this method was reported [95].

**Scheme 26:**
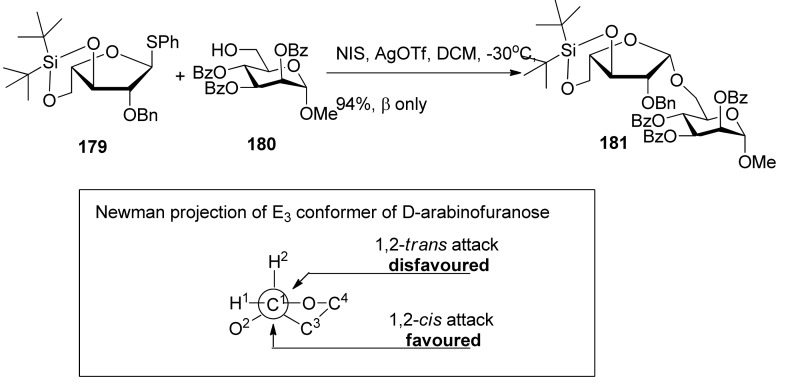
The β-selective furanosylation with 3,5-*O*-di-*tert-*butylsilylene-protected arabinosyl donor **178**.

**Figure 7:**
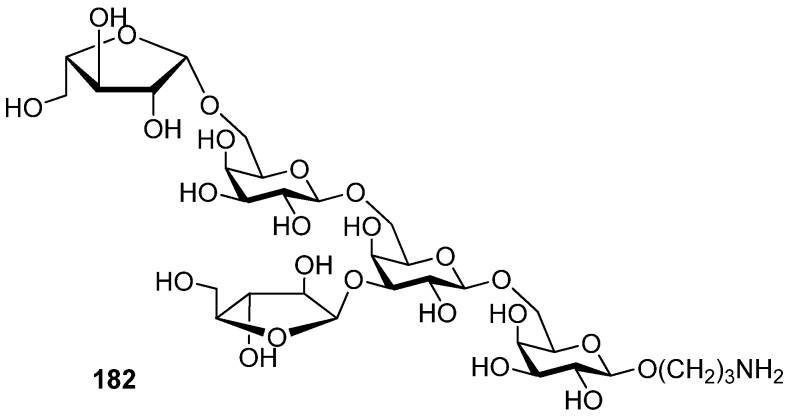
A fragment of arabinogalactans in the primary plant cell wall.

3,5-*O*-Di-*tert-*butylsilylene group was used to construct β-arabinofuranosides (Scheme 26) [96], an important constituent of microbial and plant polysaccharides. It is difficult to introduce this kind of 1,2-*cis*-furanosides due to the weak anomeric effects and flexibilities of furan rings. Only indirect protocols had been reported previously [97]. This protecting group could lock the arabinosyl donor in the low-energy conformation *E_3_*, in which nucleophilic attack from the β face was favored. As a result, a range of glycosylations of donor **179** with primary and secondary glycosyl acceptors gave the corresponding glycosides with excellent β*-*selectivity. Next, this methodology was successfully used to prepare compound **182**, a fragment of arabinogalactans in the primary plant cell wall (Figure 7). In order to probe the biosynthesis of arabinan-containing polysaccharides, Lowary also used this methodology to synthesize a docosanasaccharide and an octadecasaccharide in a highly convergent manner [98].

**Scheme 27:**
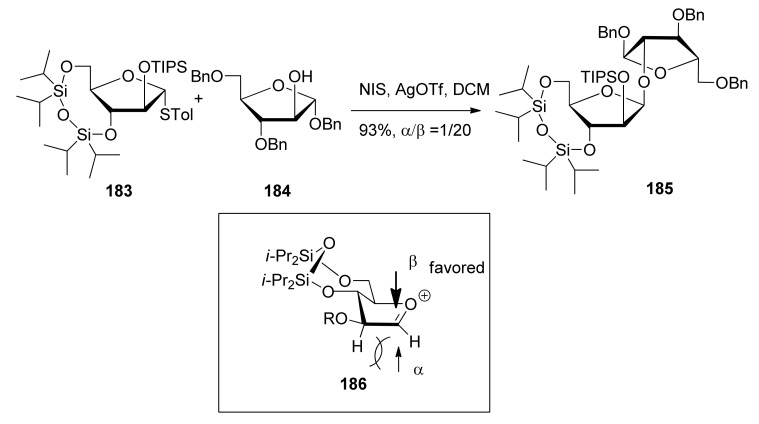
The β-selective furanosylation with 3,5-*O*-tetraisopropyldisiloxanylidene-protected donor.

β-(1,2-*Cis*)-selective furanosylation using 3,5-*O*-tetraisopropyldisiloxanylidene (TIPDS) as conformation-constraining protecting group was explored by Ito and coworkers, revealing good β-selectivity (Scheme 27) [99,100]. 3,5-*O*-Tetraisopropyldisiloxanylidene could lock the arabinofuranosyl donor **183** in the low-energy conformation *E_3_*, in which nucleophilic attack inside the envelope was favored [101]. Molecular modeling studies of these glycosylated products showed the total energy of the β-linked product was lower than that of the α-isomer, suggesting that the formation of the β-isomer was the thermodynamically favored process. In the global minimum structure, the β-product had pseudoaxially oriented glycosidic linkages, which may be favorable in light of the anomeric effect.

### 3.4. Other conformation-constraining protecting groups

**Scheme 28:**
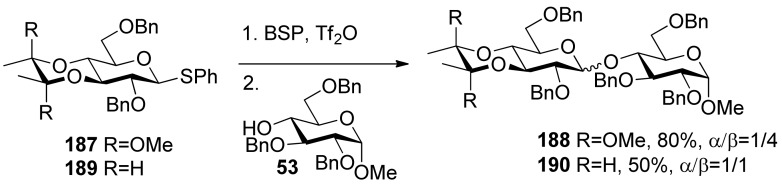
Comparision of donors **187** and **189** in glycosylation.

**Figure 8:**
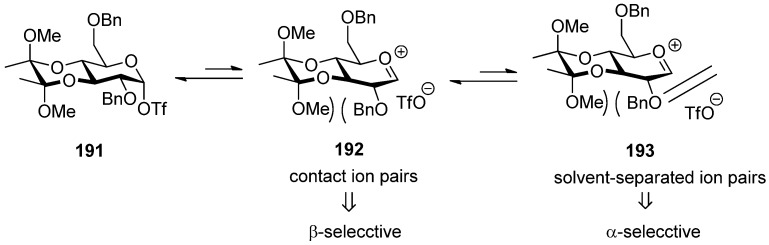
The influence of the 3,4-O-bisacetal group onglycosylation selectivity.

The 3,4-*O*-bisacetal protecting system was used for β-selective glucosylation (Scheme 28). The donor **187 **with a 3,4-*O*-bisacetal showed moderate β-selectivity but higher than that with the more electronically disarmed corresponding 3,4-*O*-carbonate [102], which showed that conformational factors could be more important than electronic factors here. According to Crich’s mechanistic hypothesis for the glycosyl triflate-based glycosylations, the explanation could be found in the equilibria shown in Figure 8, which are similar to benzylidene-directed β-mannosylation. When the covalent triflate **191**collapses to the contact ion pair **192** with its glycosyl oxacarbenium ion in a conformation approximating a *^4^H_3_* half-chair, the C2–O2 bond necessarily rotates down below the nominal pyranose plane leading to an increased steric interaction with the methoxy group of the bisacetal. It is this increased steric interaction that destabilizes the glycosyl cation sufficiently to bring about the observed β-selectivity. In donor **189** there is no longer a destabilizing steric interaction between the O2 protecting group and the cyclic protecting group spanning O3 and O4, resulting in anomeric mixtures.

**Scheme 29:**
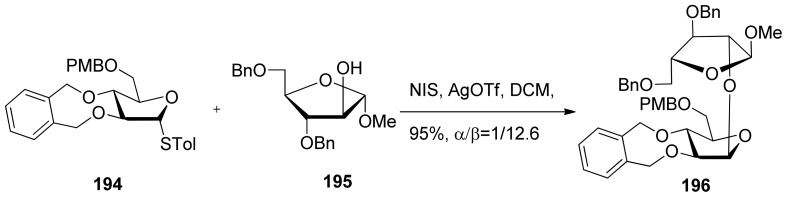
β-Arabinofuranosylation of 2,3-*O*-xylylene-protected arabinofuranosyl donor **194**.

Conformationally restricted 2,3-*O*-xylylene-protected arabinofuranosyl donors were also reported in the stereoselective β-arabinofuranosylation (Scheme 29) [103]. It was found that the stereoselectivity of glycosylations was dependent on the reaction conditions, and the use of a 5-*O*-PMB-type donor **194** was essential for relative good β-selectivity. The detailed mechanism was not checked.

## 4. Conclusions

Protecting groups play important roles in carbohydrate chemistry. As many kinds of protecting groups have been explored and used in recent years, the synthesis of oligosaccharides has made great progress, which will further benefit the development of glycobiology. However, current protecting groups are not ideal in many cases, and new protecting groups that enhance the glycosylation reactivity and control the stereochemistry outcomes of glycosylations are still needed. At present, some mechanism issues involving how protecting groups influence glycosylations are still ambiguous. The resolution of these problems will greatly promote the advances in stereocontrolled oligosaccharide synthesis.
